# T Cell Energy Metabolism Is a Target of Glucocorticoids in Mice, Healthy Humans, and MS Patients

**DOI:** 10.3390/cells12030450

**Published:** 2023-01-30

**Authors:** Leonie Meyer-Heemsoth, Katja Mitschke, Jasmina Bier, Konstantin Schütz, Andreas Villunger, Tobias J. Legler, Martin S. Weber, Fred Lühder, Holger M. Reichardt

**Affiliations:** 1 Institute for Cellular and Molecular Immunology, University Medical Center Göttingen, 37073 Göttingen, Germany; 2 Institute for Neuroimmunology and Multiple Sclerosis Research, University Medical Center Göttingen, 37075 Göttingen, Germany; 3 Department of Neurology, University Medical Center Göttingen, 37075 Göttingen, Germany; 4 Institute of Developmental Immunology, Medical University of Innsbruck, 6020 Innsbruck, Austria; 5 Department of Transfusion Medicine, University Medical Center Göttingen, 37075 Göttingen, Germany; 6 Institute of Neuropathology, University Medical Center Göttingen, 37075 Göttingen, Germany; 7 Fraunhofer-Institute for Translational Medicine and Pharmacology ITMP, 37075 Göttingen, Germany

**Keywords:** glucocorticoids, T cells, multiple sclerosis, metabolism

## Abstract

Glucocorticoids (GCs) are used to treat inflammatory disorders such as multiple sclerosis (MS) by exerting prominent activities in T cells including apoptosis induction and suppression of cytokine production. However, little is known about their impact on energy metabolism, although it is widely accepted that this process is a critical rheostat of T cell activity. We thus tested the hypothesis that GCs control genes and processes involved in nutrient transport and glycolysis. Our experiments revealed that escalating doses of dexamethasone (Dex) repressed energy metabolism in murine and human primary T cells. This effect was mediated by the GC receptor and unrelated to both apoptosis induction and Stat1 activity. In contrast, treatment of human T cells with rapamycin abolished the repression of metabolic gene expression by Dex, unveiling mTOR as a critical target of GC action. A similar phenomenon was observed in MS patients after intravenous methylprednisolon (IVMP) pulse therapy. The expression of metabolic genes was reduced in the peripheral blood T cells of most patients 24 h after GC treatment, an effect that correlated with disease activity. Collectively, our results establish the regulation of T cell energy metabolism by GCs as a new immunomodulatory principle.

## 1. Introduction

Glucocorticoids (GCs) are widely used to treat inflammatory diseases due to their diverse and potent effects on the immune system [1]. T cells play a particularly important role as targets of GC activity as demonstrated by the analysis of mouse models where repression of T cells rather than other immune cell subsets was found to be essential for therapeutic activity [2,3,4]. GCs induce T cell apoptosis, which proceeds via the intrinsic pathway and depends on the T cells’ activation state [5,6,7,8]. In addition, they also regulate the expression of various cytokines, chemokines, and cell adhesion receptors [9,10,11]. While some of these effects rely on the DNA-binding capacity of the GC receptor (GR), others are mediated by the interaction of the GR with transcription factors such as AP-1 or NF-κB [12]. Beyond these genomic activities, the GR also impacts cytosolic signaling molecules, thereby altering, for instance, T cell shape and mobility [13]. In MS patients and the corresponding animal model experimental autoimmune encephalomyelitis (EAE), it was uncovered that GCs improve disease symptoms mainly by redirecting the migration of T cells in a CXCR4-dependent manner rather than by inducing their apoptosis [11,14]. This observation highlights that the relevance of the individual immunomodulatory mechanisms of GCs must be consistently questioned.

T cells consume little energy in their resting state. Following activation, however, they require large amounts of ATP as well as metabolic intermediates such as nucleic acids (for DNA synthesis), amino acids (for protein synthesis), NADPH, and acetyl-CoA within a relatively short time frame [15,16,17]. To fulfill these needs, T cells switch their metabolism from oxidative phosphorylation (OXPHOS) to glycolysis, which is a relatively inefficient pathway but instead provides energy and metabolic intermediates in a very fast manner [18,19,20]. Enforcement of the glycolytic pathway in activated T cells results in a conversion of pyruvate to lactate and its subsequent excretion, which is accompanied by an increased gene expression and surface localization of metabolite transporters necessary to satisfy the T cells’ large demand for glucose and amino acids. These effects are mainly mediated by CD28 signaling, which activates the mTOR (mechanistic target of rapamycin) pathway [21,22], thereby leading to an upregulation of Glut1 (glucose transporter 1), enhanced glucose import, and increased expression of various metabolic enzymes [23,24]. Hence, impaired mTOR signaling is associated with a lower expression of Glut1 and diminished glucose uptake [25]. The phosphorylation of signal transducer and activator of transcription (Stat) proteins induced by cytokines and hormones regulate T cell activity as well, but their involvement in the control of energy metabolism has not been explored [26,27,28]. It is noteworthy that OXPHOS still operates in activated T cells despite the metabolic switch, although at a reduced level, and thereby supports the oxidation of glucose, fatty acids, and glutamine [15,16]. While the relevance of immunometabolism and its quick adaptation to current needs is well established, its regulation by GCs remains poorly understood [29]. There are some studies on selected aspects of this process in specialized cell types such as regulatory T cells [30], the mitochondrial metabolism in myeloid-derived suppressor cells in the context of thrombocytopenia [31], and the impact of GCs on CD8^+^ T cells involved in anti-tumor responses [32,33]. Furthermore, it was found that the inhibition of mTOR in myeloid cells blocked the anti-inflammatory activity of the GR, unveiling a link between both factors [34]. The general effects of GCs on metabolic processes, however, have not been addressed up to now.

Here, we hypothesized that the energy metabolism of T cells is a crucial rheostat of the immunomodulatory activity of GCs, partially explaining their suppressive effects on T cell activation, differentiation, and effector functions. By investigating murine T cells as well as human T cells from both healthy individuals and MS patients, we obtained new insights into the mode of GC action, which are expected to provide improved treatment efforts in the future.

## 2. Materials and Methods

### 2.1. Animal Experimentation

All mouse strains used in this work were described previously and bred in our animal facilities either in Göttingen, Germany or Innsbruck, Austria: C57BL/6 wild-type (wt) mice, *Nr3c1^tm2Gsc^*Tg^(Lck-cre)1Cwi^ C57BL/6 mice lacking the GR in T cells (designated GR^lck^), *Nr3c1^tm2Gsc^* C57BL/6 littermate controls (designated GR^flox^), *Tg(Vav-BCL2)1Jad* mice overexpressing Bcl-2 in hematopoietic cells (designated Bcl-2 tg) as well as their littermate controls [2,11]. All mice were kept in individually ventilated cages under specific pathogen-free conditions and supplied with food and water ad libitum.

### 2.2. Isolation and Culture of Murine T Cells

T cells were isolated from lymph nodes and spleen using the EasySep^®^ Mouse T Cell Isolation Kit (StemCell Technologies, Cologne, Germany) according to the manufacturer’s protocol, diluted in RPMI medium with 10% FCS and 1% Penicillin/Streptomycin (RPMI^+^) at a density of 1 × 10^6^ cells/mL and seeded in 24-well plates with 1 ml per well. T cell purity was routinely >92% as determined by flow cytometry. T cells were activated for up to 24 h by incubation with soluble mCD3ε (1 μg/mL; clone 145-2C11) and mCD28 (5 μg/mL; clone 37.51) antibodies (both from BioLegend; Uithoorn, The Netherlands) at 37 °C and 5% CO_2_. Dexamethasone (Ratiopharm, Ulm, Germany) was added to cell cultures at different concentrations during the entire incubation time.

### 2.3. Isolation and Culture of Human T Cells

PBMCs were isolated from buffy coats of healthy blood donors by density gradient centrifugation as previously described [35]. Then, T cells were purified from PBMCs using the EasySep^®^ Human T cell Isolation Kit (StemCell Technologies) according to the manufacturer’s protocol. The purity of T cell preparations was generally around 98% as tested by flow cytometry. T cells were cultured in 48-well plates at a density of 3 × 10^6^ cells/mL in 250 µL RPMI^+^ medium. T cell activation was achieved by first coating the wells with hCD3ε (1 µL/mL; clone OKT3) antibody for 2 h at 37 °C and then adding soluble hCD28 (5 µg/mL; clone CD28.2) antibody to the culture medium (BioLegend). T cells were incubated for 20 h at 37 °C and 5% CO_2_. Fludarabine was added at 50 µg/mL (diluted from a 50 mg/mL stock solution in DMSO; Selleckchem, Houston, TX, USA) and rapamycin at 20 nM (diluted from a 1 mM stock solution in EtOH; Sigma-Aldrich, Taufkirchen, Germany). Both inhibitors were present during the entire culture period.

### 2.4. Quantitative RT-PCR

Total RNA from T cells was either isolated using TRIzole reagent (ThermoFisher, Osterode, Germany) in combination with chloroform and isopropanol (Carl Roth, Karlsruhe, Germany) or with the help of the *Quick*-RNA Miniprep Kit (Zymo Research, Irvine, CA, USA). Then, 0.5 to 1 µg was reverse-transcribed into cDNA with the RevertAid First Strand cDNA Synthesis Kit (ThermoFisher) or with the iScript Kit (Bio-Rad, Munich, Germany). Quantitative RT-PCR (RT-qPCR) analysis was performed on an ABI 7500 Instrument (ThermoFisher) by employing the respective SYBR Green Master Mix. The housekeeping gene *Hprt* was used for normalization of gene expression in murine cells, and *18SRNA* was used in the case of human cells. Relative expression levels of target genes were calculated with the ∆∆Ct method. All primers were synthesized by Metabion (Planegg, Germany), and their sequences are listed in Supplementary Table S1.

### 2.5. Flow Cytometry

Extracellular staining of T cells was performed with monoclonal antibodies obtained from BioLegend: mCD4 (H129.19), mCD8α (53–6.7), hCD3ε (HIT3A), hCD4 (OKT4), hCD8α (HIT8a), hCD69 (FN50). Intracellular staining of phosphorylated Stat1 and mTOR was achieved using reagents from BD Biosciences (Heidelberg, Germany) and antibodies from ThermoFisher, pStat1-Tyr701 (KIKSI0803) and pmTOR-Ser2448 (MRRBY), following our published protocol [36]. The cells were either analyzed with a FACSCantoII^®^ (BD Biosciences) or a Cytoflex^®^ flow cytometer (Beckmann Coulter, Krefeld, Germany) in combination with FlowJo^®^ (version 10.7, Treestar, Ashland, OR, USA) software. Apoptosis induction in T cells was measured by incubation with FITC-labeled AnnexinV (AnxV; BioLegend) as described previously [8]. Glucose uptake by T cells was determined using a commercial kit (Cayman Chemical, Ann Arbor, MI, USA) with a fluorescently labeled deoxyglucose analog (2-NBDG). To this end, the cells were resuspended in 100 µL glucose-free medium supplemented with 150 µg/mL 2-NBDG, incubated at 37 °C for 1 h, and analyzed by flow cytometry [37].

### 2.6. Lactate Assay

Glycolytic activity of cultured T cells was investigated by quantifying the amount of L-lactate secreted into the medium with a commercially available kit (Cayman Chemical) using a fluorescence-based method as described previously [37]. Cell culture supernatants were deproteinated with metaphosphoric acid and subsequently neutralized. Then, the salts were precipitated and the samples placed in black 96-well microtiter plates. Lactate was converted to pyruvate in an enzymatic reaction, yielding a fluorescent product that was detected with an excitation wavelength of 530–540 nm and an emission wavelength of 585–595 nm using an Infinite 200 Pro reader (Tecan, Männedorf, Switzerland).

### 2.7. T Cell Isolation from MS Patients

Blood samples from MS patients with a primary or secondary progressive disease course (PPMS, SPMS) were collected at the Neurology Day Clinic of the UMG Göttingen. Patient characteristics are summarized in Supplementary Table S2. Two blood samples of 8 mL each were drawn from patients receiving high-dose intravenous methylprednisolone (IVMP) pulse therapy by venous puncture, one before and the other 24 h after the first dose of 1.000 mg MP, using Multifly^®^ canules and BD Vacutainer^®^ CPT^TM^ tubes. After centrifugation, the PBMCs were removed and washed with PBS and the T cells were purified using the EasySep^®^ Human T cell Isolation Kit (StemCell Technologies) as described above. The purity was routinely >95% with a level of activation of <5%.

### 2.8. Statistics

Data were analyzed by one-way ANOVA followed by Tukey multiple comparisons test or by unpaired *t*-test using GraphPad Prism^®^ software (version 9.4, San Diego, CA, USA). All data are depicted as the mean ± SEM. Levels of significance: n.s.: *p* > 0.05; * *p* < 0.05; ** *p* < 0.01; *** *p* < 0.001; multiplicity-adjusted *p* values are reported in the case of a one-way ANOVA.

## 3. Results

### 3.1. Dex Represses Metabolic Gene Expression in Murine T Cells in a GR-Dependent Manner

**Figure 1:**
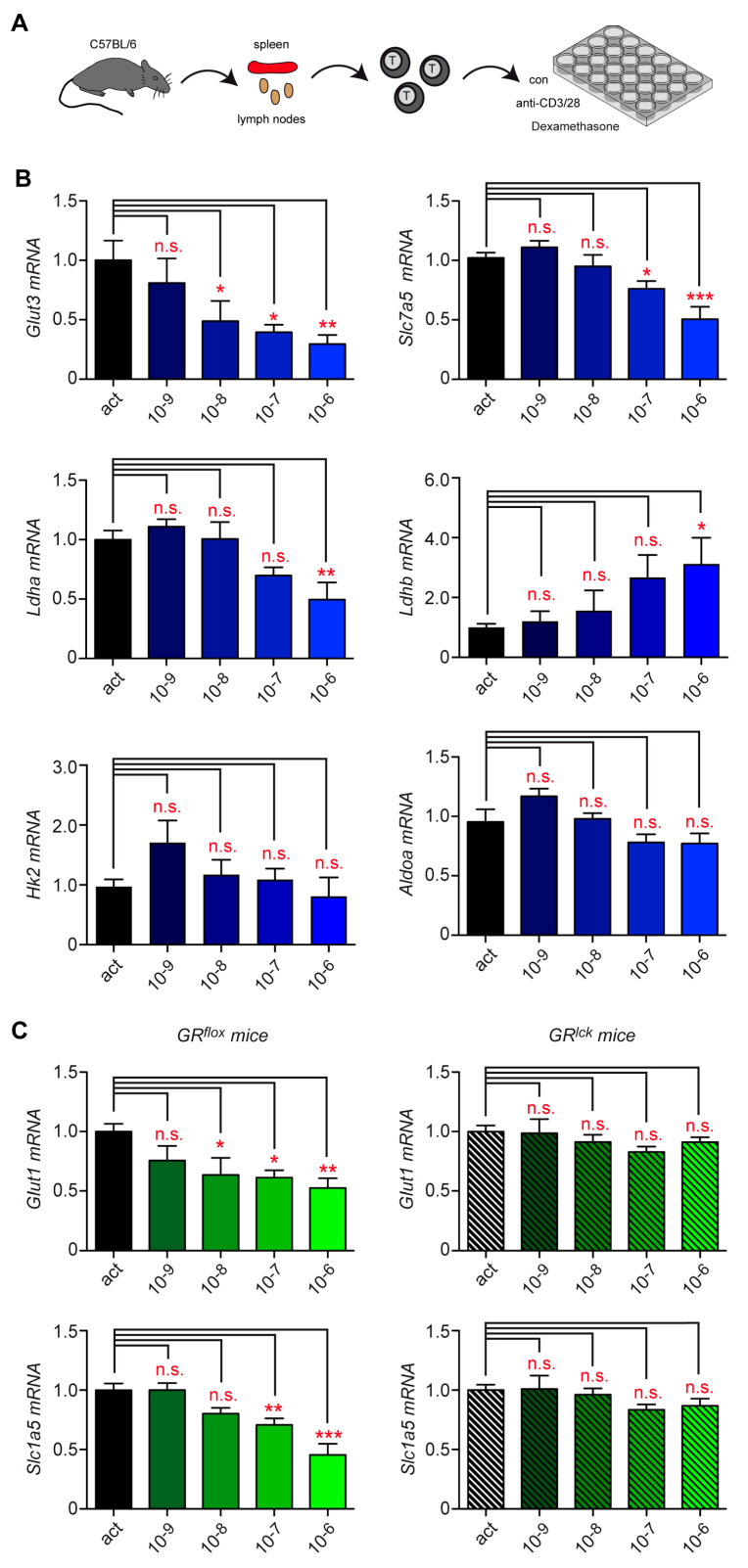
Repression of metabolic genes in murine T cells by Dex treatment in vitro. (**A**) Setup of the experimental model. (**B**) T cells were purified from spleen and lymph nodes of C57BL/6 wild-type mice and stimulated with mCD3ε and mCD28 antibodies for 24 h, either in the absence (act) or presence of 10^−9^ to 10^−6^ M Dex. Gene expression was determined by RT-qPCR. N = 7–30. (**C**) T cells were purified from GR^flox^ or GR^lck^ mice and treated as described for panel B. Gene expression was determined by RT-qPCR. N = 10–15. Relative mRNA levels were calculated by normalization to the housekeeping gene *Hprt.* Expression in activated T cells was arbitrarily set to 1. Values are depicted as the mean ± SEM. Statistical analysis was performed by one-way ANOVA followed by a multiple comparisons test. Levels of significance: * *p* < 0.05; ** *p* < 0.01; *** *p* < 0.001; n.s.: *p* > 0.05.

Initially, we used murine T cells to test the hypothesis that genes involved in energy metabolism were regulated by GCs. To this end, total T cells were isolated from secondary lymphoid organs of C57BL/6 wild-type mice and activated for 24 h using mCD3ε and mCD28 antibodies (Figure 1A). To some samples, 10^−6^ M Dex was added during the last 6, 10, or 24 h. Subsequently, we analyzed the expression of genes contributing to the rapid switch from steady-state OXPHOS to activation-induced glycolysis by RT-qPCR. These included the glucose transporters *Glut1* and *Glut3,* the amino acid transporters *Slc1a5* and *Slc7a5*, and the glycolytic enzymes *Ldha*, *Ldhb*, *Hk2*, and *AldoA*. Dex treatment for 24 h influenced the expression of most genes, although to different degrees (Supplementary Figure S1). In contrast, gene expression remained unaltered when Dex was added for shorter time periods. In the next step, we tested the effects of escalating doses of Dex for the 24 h incubation period. For this, T cells were activated using mCD3ε and mCD28 antibodies and concomitantly treated with 10^−9^ M to 10^−6^ M Dex (Figure 1A). This analysis revealed that the modulation of metabolic gene expression was influenced by GCs in a concentration-dependent manner (Figure 1B,C).

To confirm that GCs exerted their repressive activity via their bona fide receptor, we used GR^lck^ mice selectively lacking the GR in the entire T cell lineage. As exemplified for the two metabolite transporters *Glut1* and *Slc1a5*, the repressive effect of Dex on gene expression observed in wild-type GR^flox^ T cells was abolished in GR^lck^ mice (Figure 1C). In the absence of Dex, gene expression in activated T cells was similar in both genotypes (data not shown). Collectively, our findings provide the first evidence that GCs regulate critical metabolic genes in activated murine T cells in a GR-dependent manner.

### 3.2. Control of Metabolic Gene Expression by Dex Is Unrelated to Their Pro-Apoptotic Activity

**Figure 2:**
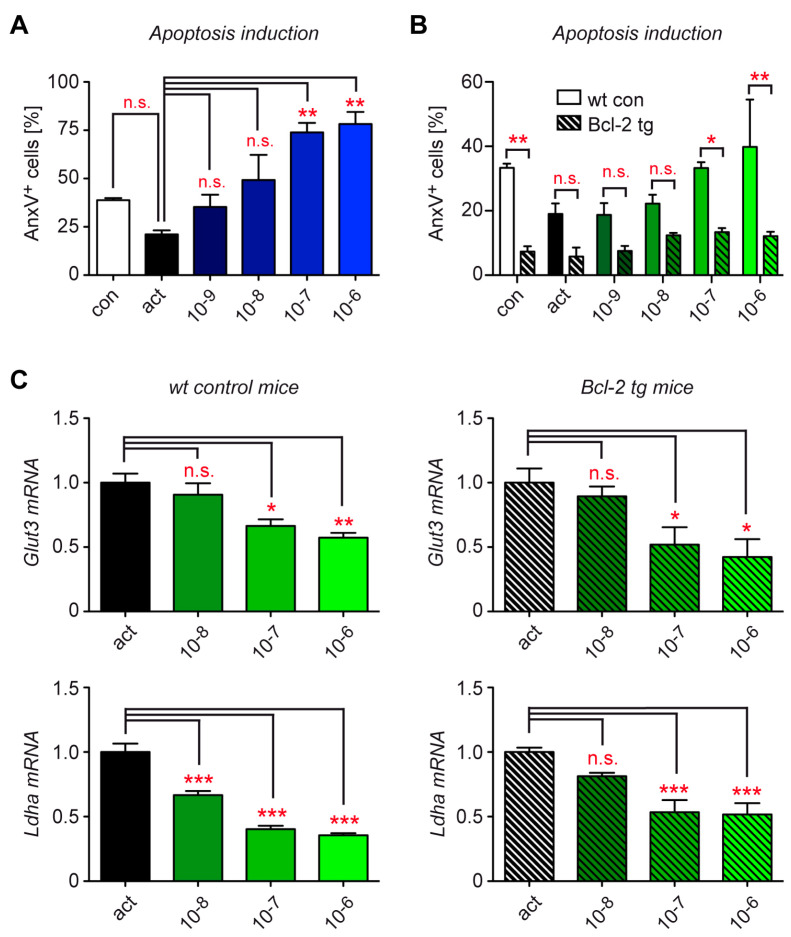
Role of apoptosis induction for the metabolic control of murine T cells by Dex in vitro. (**A**) T cells were purified from spleen and lymph nodes of C57BL/6 wild-type mice and stimulated with mCD3ε and mCD28 antibodies for 24 h in the absence (act) or presence of 10^−9^ to 10^−6^ M Dex. T cells without stimulation served as a control (con). Apoptosis induction was determined by flow cytometric analysis of AnxV^+^ cells amongst CD3^+^ CD4^+^ T cells. N = 4. (**B**) T cells were purified from wild-type (wt), control (con), or Bcl-2 tg mice. Treatment and analysis were performed as in panel A. N = 3. (**C**) T cells were obtained from wt, control, or Bcl-2 tg mice and treated as in panels A/B. Gene expression was determined by RT-qPCR. N = 5. Relative mRNA levels were calculated by normalization to the housekeeping gene *Hprt*. Expression in activated T cells was arbitrarily set to 1. Values are depicted as the mean ± SEM. Statistical analysis was performed by one-way ANOVA followed by a multiple comparisons test. Levels of significance: * *p* < 0.05; ** *p* < 0.01; *** *p* < 0.001; n.s.: *p* > 0.05.

The induction of T cell apoptosis is one of the most prominent effects of GCs. Accordingly, the addition of escalating doses of Dex during T cell activation caused extensive cell death (Figure 2A). To investigate whether the observed repression of metabolic gene expression in T cells was truly a direct effect of GCs rather than a consequence of apoptosis induction, we used transgenic mice overexpressing the anti-apoptotic molecule Bcl-2 in hematopoietic cells [11]. As expected, T cells from Bcl-2 tg mice were resistant to the pro-apoptotic activity of GCs, which is in sharp contrast to cells from their wild-type littermates (Figure 2B). When we subsequently compared the regulation of metabolic gene expression in activated T cells by Dex, we did not detect any differences between mice of both genotypes. Regardless of whether T cells were sensitive to GC-induced apoptosis or not, the expression levels of *Glut3* and *Ldha* were both reduced by escalating doses of Dex (Figure 2C). This finding confirms that GCs exert control over metabolic gene expression independently of apoptosis induction.

### 3.3. Dex Interferes with the Activation of T Cells from Healthy Human Subjects

**Figure 3:**
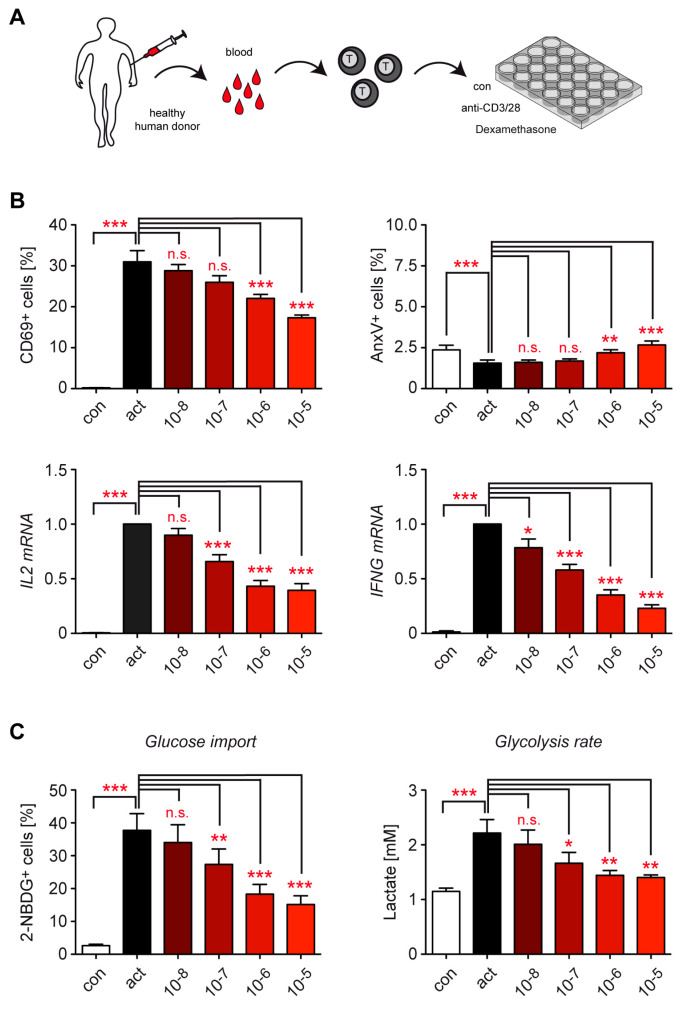
Repression of T cell functions and metabolic processes in human peripheral blood T cells by Dex in vitro. (**A**) Setup of the experimental model. (**B**) T cells were purified from buffy coats of healthy human donors and stimulated with hCD3ε and hCD28 antibodies for 20 h in the absence (act) or presence of 10^−8^ to 10^−5^ M Dex. Unstimulated T cells served as a control (con). The percentages of CD69^+^ and AnxV^+^ CD3^+^ CD4^+^ T cells were determined by flow cytometric analysis; N = 17/12. *IL2* and *IFNG* gene expression was analyzed by RT-qPCR. N = 12. Relative mRNA levels were calculated by normalization to the housekeeping gene *18SRNA*. Expression in activated T cells was arbitrarily set to 1. (**C**) T cells were purified and treated as described for panel B. Glucose import was determined by incubating the cells for 1 hr with fluorescently labeled 2-NBDG and measuring the percentage of CD3^+^ CD4^+^ T cells positively stained for 2-NBDG by flow cytometry (left panel, N = 15). The glycolysis rate was analyzed by measuring the absolute concentration of lactate in the cell culture supernatant with the help of a photometric assay (right panel, N = 4). All values are depicted as the mean ± SEM. Statistical analysis was performed by one-way ANOVA followed by a multiple comparisons test. Levels of significance: * *p* < 0.05; ** *p* < 0.01; *** *p* < 0.001; n.s.: *p* > 0.05.

Next, we switched to human peripheral blood T cells, which more closely mimic the immune system of patients harboring many antigen-experienced T cells in the circulation (Figure 3A). Stimulation with hCD3ε and hCD28 antibodies increased the percentage of CD3^+^ CD4^+^ T cells expressing the activation marker CD69 in comparison to resting control cells, an effect which was significantly diminished by the addition of 10^−8^ M to 10^−5^ M Dex (Figure 3B). Notably, a similar effect was observed for CD3^+^ CD8^+^ T cells (Supplementary Figure S2A). The increased percentage of CD69^+^ cells after T cell activation was paralleled by elevated *IL2* and *IFNG* mRNA levels in total T cells, which were both reduced by Dex (Figure 3B). Quantification of CD4^+^ as well as CD8^+^ T cells positively stained for AnnexinV (AnxV) revealed that apoptosis levels were diminished after activation but enhanced by the addition of escalating doses of Dex (Figure 3B, Supplementary Figure S2B). The degree of apoptosis induction was much lower than previously detected in murine T cells (Figure 2A) and remained <5% under all conditions (Figure 3B). This observation indicates that human peripheral blood T cells are much less sensitive to GC action than murine T cells from secondary lymphoid organs, which is also the reason why we used higher concentrations of Dex in all experiments involving human T cells compared to murine T cells. Taken together, a major influence of apoptosis on T cell metabolism can be excluded in our human model system.

### 3.4. Metabolite Transport and Glycolysis in Human T Cells Are Repressed by Dex

The rapid switch from OXPHOS to glycolysis in the course of T cell activation is accompanied by strongly enhanced glucose uptake. To investigate whether this critical step is already influenced by GCs, we initially tested the transport capacity of activated human peripheral blood CD4^+^ T cells for glucose using a flow cytometric assay with 2-NBDG, a fluorescent glucose analog that cannot be metabolized and thus accumulates in the cell. It turned out that CD4^+^ T cells increased glucose import following activation, which was repressed by escalating doses of Dex (Figure 3C). It is noteworthy that a similar finding was made for CD8^+^ T cells from peripheral blood (Supplementary Figure S2C). To further determine the impact of Dex on the glycolytic activity of T cells, we measured the amount of lactate present in cell culture supernatants after 20 h using a photometric assay. Activated human T cells excreted higher amounts of lactate than resting T cells, which was inhibited by escalating doses of Dex (Figure 3C).

**Figure 4:**
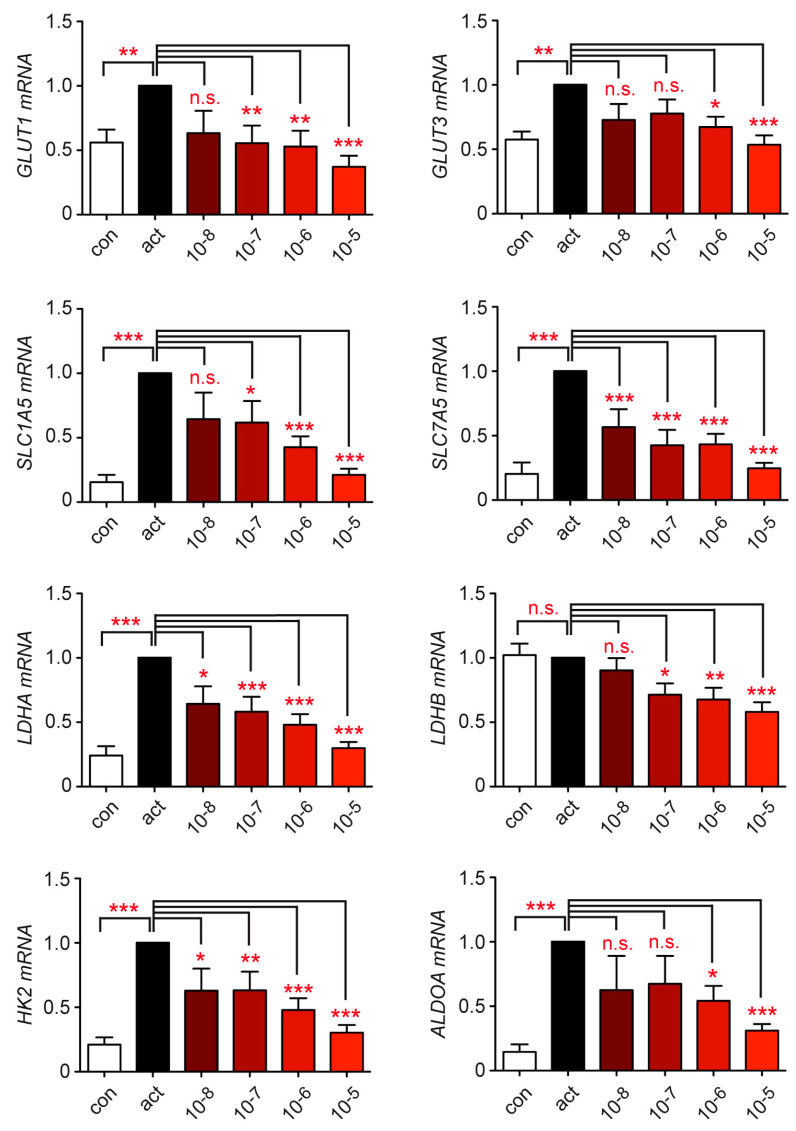
Repression of metabolic genes in human peripheral blood T cells by Dex treatment in vitro. T cells were purified from buffy coats of healthy human donors and stimulated with hCD3ε and hCD28 antibodies for 20 h in the absence (act) or presence of 10^−8^ to 10^−5^ M Dex. Unstimulated T cells served as a control (con). Gene expression was determined by RT-qPCR. N = 9–12. Relative mRNA levels were calculated by normalization to the housekeeping gene *18SRNA*. Expression in activated T cells was arbitrarily set to 1. Values are depicted as the mean ± SEM. Statistical analysis was performed by one-way ANOVA followed by a multiple comparisons test. Levels of significance: * *p* < 0.05; ** *p* < 0.01; *** *p* < 0.001; n.s.: *p* > 0.05.

Finally, we investigated the gene expression of the glucose and amino acid transporters *GLUT1*, *GLUT3*, *SLC1A5*, and *SLC7A5* as well as the glycolytic enzymes *LDHA*, *LDHB*, *HK2*, and *ALDOA*. T cell activation for 20 h increased the mRNA levels of most genes, while concomitant treatment with escalating doses of Dex strongly repressed them (Figure 4). It is noteworthy that the two genes encoding lactate dehydrogenase isoforms, which fulfil opposite functions in glycolysis, were regulated differently. *LDHA* was induced by T cell activation whereas the gene expression of *LDHB* remained unaltered. Dex repressed both genes, although the effect was more profound for *LDHA* than *LDHB*, an observation which is compatible with the overall reduced production of lactate under this condition (Figure 3C and Figure 4). In summary, Dex represses glucose and amino acid import into activated T cells and reduces the expression of glycolytic enzymes necessary for the conversion of glucose into lactate. It is thus plausible that the immunosuppressive activity of Dex at least partially depends on effects exerted on genes and processes involved in the energy metabolism of T cells.

### 3.5. Stat1 Is Dispensable for the Inhibitory Effect of GCs on Metabolic Gene Expression in T Cells

**Figure 5:**
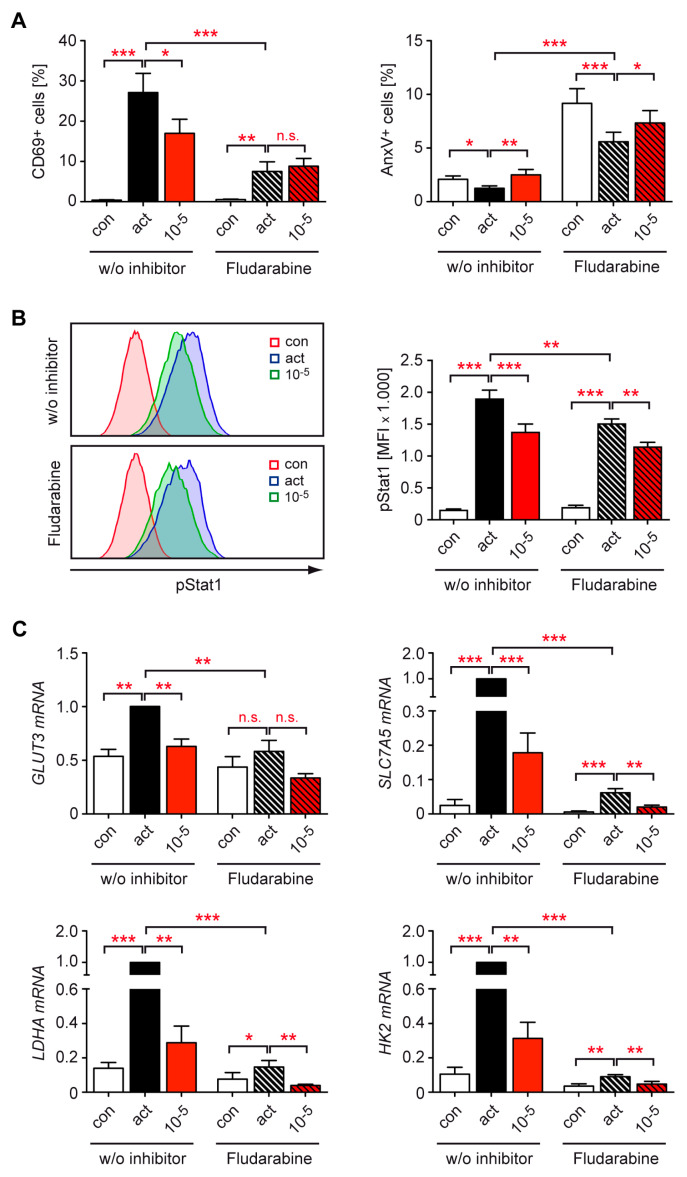
Role of Stat1 signaling for the repressive activity of Dex on T cell function and metabolic gene expression in human peripheral blood T cells. (**A**) T cells were purified from buffy coats of healthy human donors and stimulated with hCD3ε and hCD28 antibodies for 20 h in the absence (act) or presence of 10^−5^ M Dex. Unstimulated T cells served as a control (con). To study the role of Stat1, fludarabine was added at a concentration of 50 µg/mL to the same experimental setup. The percentages of CD69^+^ and AnxV^+^ CD3^+^ CD4^+^ T cells were determined by flow cytometric analysis. N = 10/12. (**B**) T cells were treated as in panel A. After extracellular staining for CD3, CD4, and CD69, the cells were fixed, permeabilized, and then intracellularly stained for pStat1 (Tyr701). Analysis was performed by flow cytometry. Exemplary overlay histograms of pStat1 stainings of CD4^+^ T cells (con) or CD4^+^ CD69^+^ T cells (act, 10^−5^ M Dex) are depicted in the left panel. The mean fluorescence intensity (MFI) of pStat1 for CD4^+^ T cells (con) or CD4^+^ CD69^+^ T cells (act, 10^−5^ M Dex) is depicted in the right panel. N = 6. (**C**) Expression of metabolic genes was determined by RT-qPCR. N = 7. Relative mRNA levels were calculated by normalization to the housekeeping gene *18SRNA*. Expression in activated T cells was arbitrarily set to 1. Values are depicted as the mean ± SEM. Statistical analysis was performed by one-way ANOVA followed by a multiple comparisons test. Levels of significance: * *p* < 0.05; ** *p* < 0.01; *** *p* < 0.001; n.s.: *p* > 0.05.

Multiple transcription factors are well known to be involved in the control of T cell activity including Stat1, which has previously been shown to be regulated by GCs [38,39]. In an attempt to identify the mechanism underlying the repression of metabolic gene expression by Dex, we activated human T cells in the presence of the Stat1 inhibitor fludarabine. Under this condition, the percentage of activated CD69^+^ CD4^+^ T cells was significantly reduced in comparison to control cultures, and treatment with 10^−5^ M Dex was no longer able to diminish T cell activation (Figure 5A). In contrast, the percentage of AnxV^+^ apoptotic CD4^+^ T cells was overall increased by fludarabine, and the pro-apoptotic effect of Dex was retained (Figure 5A).

**Figure 6:**
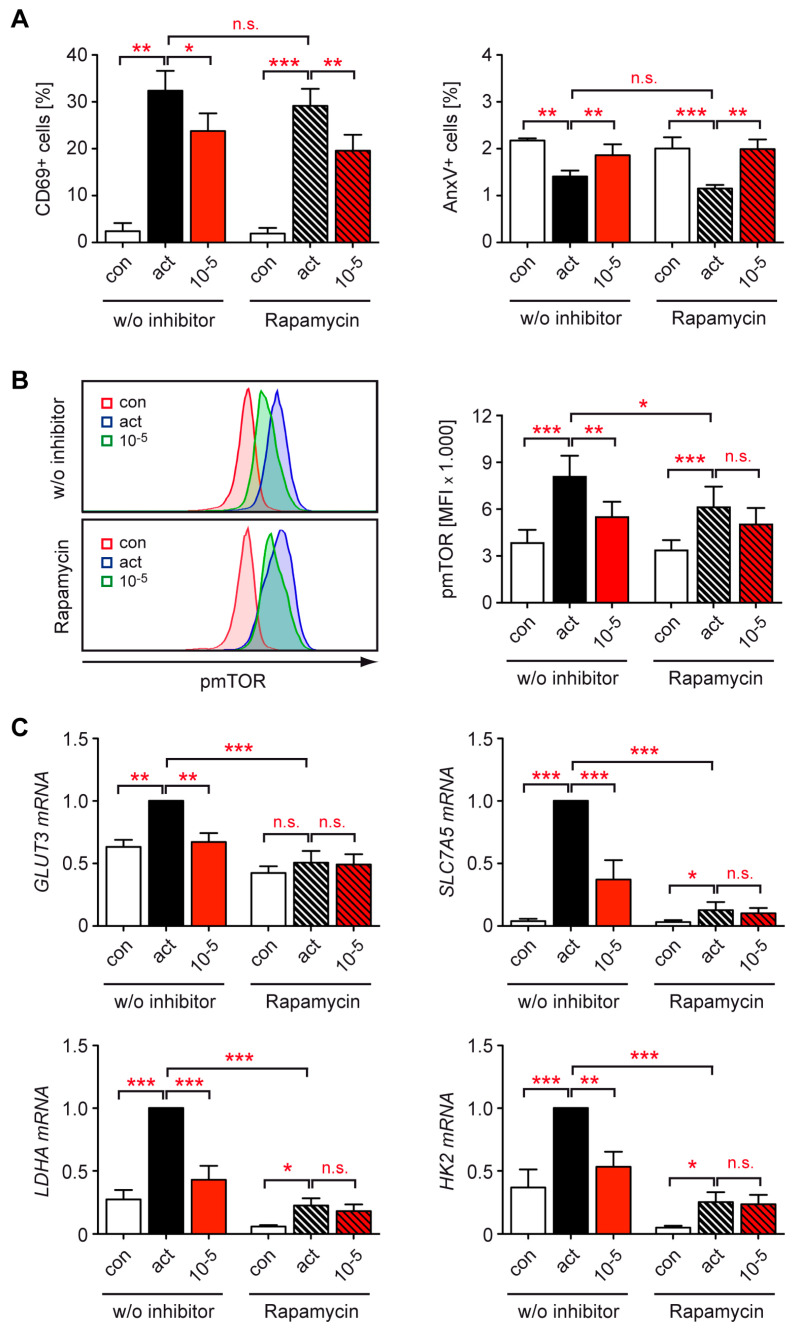
Role of mTOR signaling for the repressive activity of Dex on T cell function and metabolic gene expression in human peripheral blood T cells. (**A**) T cells were purified from buffy coats of healthy human donors and stimulated with hCD3ε and hCD28 antibodies for 20 h in the absence (act) or presence of 10^−5^ M Dex. Unstimulated T cells served as a control (con). To study the role of mTOR, rapamycin was added at a concentration of 20 nM to the same experimental setup. The percentages of CD69^+^ and AnxV^+^ CD3^+^ CD4^+^ T cells were determined by flow cytometric analysis. N = 9/3. (**B**) T cells were treated as in panel A. After extracellular staining for CD3, CD4, and CD69, the cells were fixed, permeabilized, and intracellularly stained for pmTOR (Ser2448). Analysis was performed by flow cytometry. Exemplary overlay histograms of pmTOR stainings of CD4^+^ T cells (con) or CD4^+^ CD69^+^ T cells (act, 10^−5^ M Dex) are depicted in the left panel. The mean fluorescence intensity (MFI) of pmTOR for CD4^+^ T cells (con) or CD4^+^ CD69^+^ T cells (act, 10^−5^ M Dex) is depicted in the right panel. N = 6. (**C**) Gene expression was determined by RT-qPCR. N = 7. Relative mRNA levels were calculated by normalization to the housekeeping gene *18SRNA*. Expression in activated T cells was arbitrarily set to 1. Values are depicted as the mean ± SEM. Statistical analysis was performed by one-way ANOVA followed by a multiple comparisons test. Levels of significance: * *p* < 0.05; ** *p* < 0.01; *** *p* < 0.001; n.s., non-significant (*p* > 0.05).

To investigate the signaling capacity of Stat1, we analyzed its phosphorylation at residue Tyr701. Activation strongly increased pStat1 levels in CD4^+^ T cells, albeit to a lesser degree in the presence of fludarabine. Dex had a moderate but statistically significant inhibitory effect on Stat1 phosphorylation in activated cells under both conditions (Figure 6B, Supplementary Figure S3). Subsequently, we set out to analyze whether the repressive activity of Dex on metabolic gene expression was dependent on Stat1 signaling. Upregulation of *GLUT3*, *SLC7A5*, *LDHA*, and *HK2* mRNA levels in activated T cells was significantly blunted by fludarabine treatment as expected, but Dex was still able to reduce the expression of all four genes despite the inhibition of Stat1 (Figure 5C). We thus conclude that Stat1 plays a role in T cell activation, apoptosis induction, and energy metabolism, but it does not seem to represent a relevant target of the repressive activity of Dex in the latter process.

### 3.6. Dex Represses T Cell Metabolism by Controlling mTOR Signaling

T cell activation involving CD28 engagement stimulates mTOR signaling, which results in enhanced glucose transport and increased glycolytic activity [40]. Hence, we hypothesized that Dex may exert some of its repressive activity on the metabolic program of T cells by interacting with mTOR. To address this issue, human T cells were stimulated for 20 h in the absence or presence of the specific mTOR inhibitor rapamycin and treated with 10^−5^ M Dex. Rapamycin influenced neither the percentage of CD69^+^ CD4^+^ T cells nor the level of apoptosis, and the regulation of these processes by Dex was similar under both conditions. From this finding, we conclude that mTOR signaling is dispensable for these two GC activities (Figure 6A).

As expected, T cell stimulation induced mTOR phosphorylation at residue Ser2448, which reflects an activation of this signaling pathway, although the effect was less pronounced in the presence of rapamycin (Figure 6B, Supplementary Figure S4). Under control conditions, Dex treatment reduced pmTOR levels in activated CD4^+^ T cells to almost basal levels. In the presence of rapamycin, however, its inhibitory effect on mTOR phosphorylation was largely lost (Figure 6B, Supplementary Figure S4). Finally, we analyzed metabolic gene expression by RT-qPCR and found that rapamycin interfered with the upregulation of *GLUT3*, *SLC7A5*, *LDHA*, and *HK2* mRNA levels in activated T cells, reflecting its inhibitory effect on mTOR signaling. Importantly, however, Dex was no longer able to inhibit the expression of these genes in the presence of rapamycin (Figure 6C). This observation suggests that mTOR signaling is an important target of Dex action and essential for its repressive activity on T cell metabolism.

### 3.7. Control of T Cell Metabolism in MS Patients Receiving IVMP Therapy

**Figure 7:**
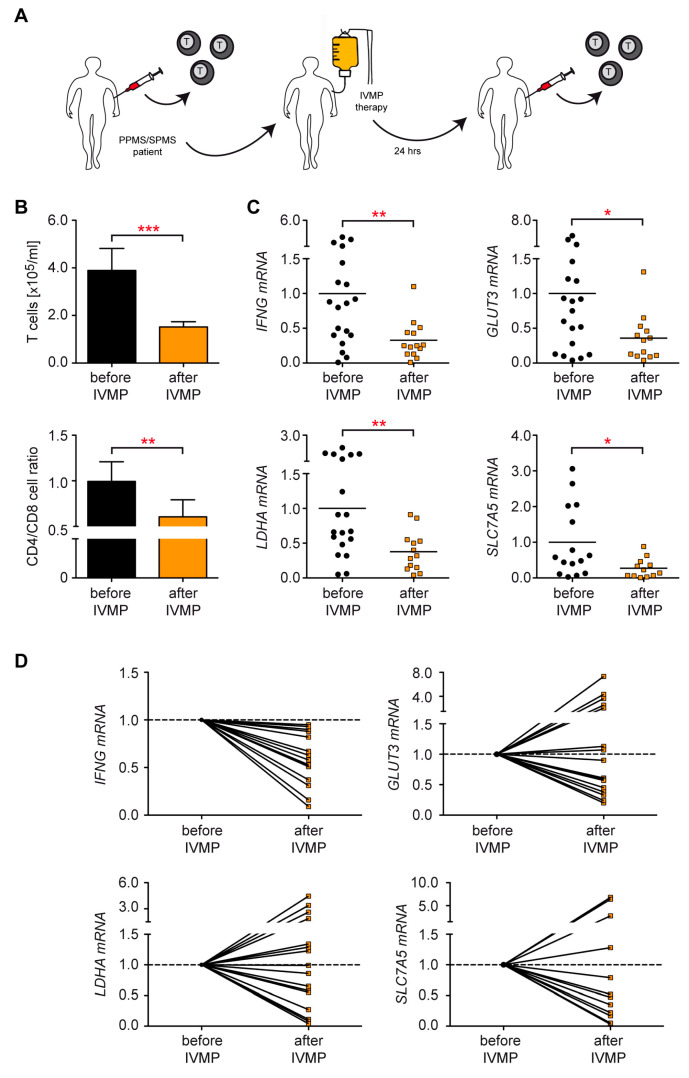
Comparison of metabolic gene expression in human peripheral blood T cells obtained from MS patients before and after IVMP therapy. (**A**) Setup of the experimental model. (**B**) T cells were purified from blood collected from PPMS and SPMS patients immediately before IVMP therapy and 24 h later; stained with CD3, CD4, and CD8 antibodies; and analyzed by flow cytometry. Absolute numbers of CD3^+^ T cells per ml of blood (upper panel) and the ratio between CD4^+^ and CD8^+^ T cells (lower panel) are depicted as the mean ± SEM. N = 18/17. (**C**) T cells were purified from blood samples as described for panel B. Gene expression was determined by RT-qPCR, and relative mRNA levels were calculated by normalization to the housekeeping gene *18SRNA.* N = 12–19. The average expression in T cells isolated before IVMP administration was arbitrarily set to 1 for each gene. Each dot corresponds to one patient. Statistical analysis in panels B/C was performed by one-way ANOVA followed by a multiple comparisons test. Levels of significance: * *p* < 0.05; ** *p* < 0.01; *** *p* < 0.001. (**D**) Presentation of the RT-qPCR results from panel C individually for each patient. N = 13–16. Gene expression in T cells isolated before IVMP infusion was arbitrarily set to 1 for each individual patient, and the relative gene expression after therapy was calculated in relation to it.

Intravenous administration of 1.000 mg methylprednisolone (IVMP) is a standard therapy to manage acute disease exacerbations in relapsing–remitting MS patients [41]. However, cyclic IVMP pulse therapy consisting of three consecutive doses of 1.000 mg may also be considered for MS patients with a primary (PPMS) or secondary (SPMS) progressive disease course as an individual treatment [42,43]. Such an approach is worth considering under the aspect of symptomatic optimization based on clinical data but without evidence from larger clinical trials [44,45]. To investigate the T cells’ response to this therapeutic regimen, we collected blood samples from a total of 12 PPMS and 17 SPMS patients, both before the first GC administration and 24 h later (Figure 7A). T cell numbers were reduced after IVMP therapy. Furthermore, the ratio between CD4^+^ and CD8^+^ T cells was shifted towards the latter ones (Figure 7B). These findings indicate that the efficacy of IVMP in this therapeutic regimen can be expected to depend at least in part on the modulation of T cell function.

Subsequently, we analyzed gene expression in T cells purified from the patient’s peripheral blood. IVMP therapy not only reduced the mRNA levels of *IFNG* but also *GLUT*3, *SLC7A*5, and *LDHA*, confirming that therapeutically applied GCs repress genes involved in the energy metabolism of T cells in MS patients with a progressive disease course (Figure 7C). Considering the previously described immunological differences between PPMS and SPMS patients [46], we additionally analyzed gene expression in both disease entities separately. Importantly, all four genes were repressed after IVMP regardless of the MS subtype, although statistical significance was only achieved for SPMS patients (Supplementary Figure S5). To further support our conclusion, we individually compared *IFNG*, *GLUT*3, *SLC7A*5, and *LDHA* gene expression before and after IVMP in each patient. *IFNG* expression was reduced in every single patient as expected, although to a different degree (Figure 7D). The metabolic genes were repressed by the infused MP in many patients too, although not in all of them (Figure 7D). We are confident that the observed alterations in gene expression are independent of the altered ratio between CD4^+^ and CD8^+^ T cells after IVMP (Figure 7B) since our previous analyses of T cells from healthy individuals indicated that GC effects in both T cell subtypes were similar (Figure 3, Supplementary Figure S2). Collectively, our results demonstrate that GC administration reduces the expression of genes involved in the energy metabolism of T cells in PPMS and SPMS patients, which possibly contributes to the clinical benefit of this therapy.

### 3.8. Correlation of Clinical Parameters with the Metabolic Control of T Cells by IVMP

**Figure 8:**
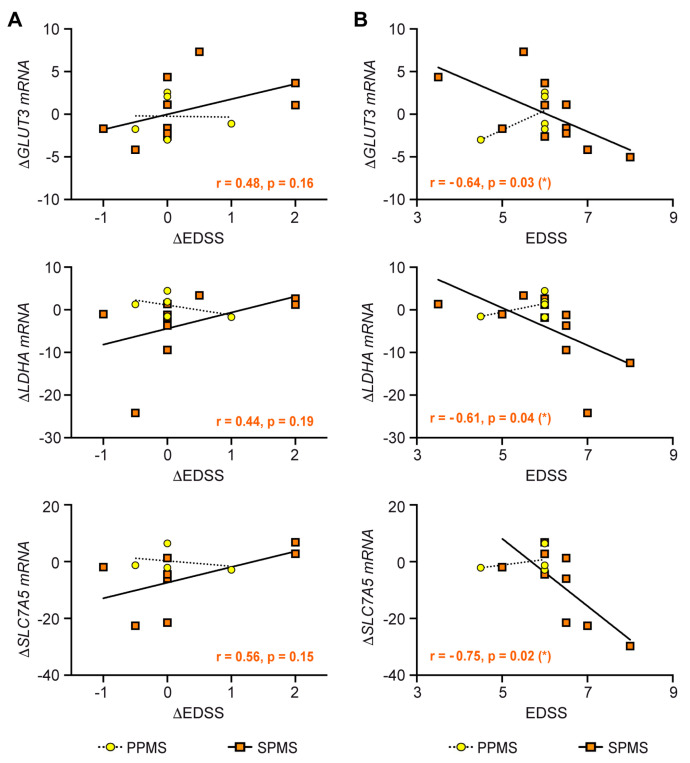
Correlation between changes in metabolic gene expression in human peripheral blood T cells from PPMS and SPMS patients during IVMP therapy and clinical parameters. (**A**) For each patient the ∆EDSS score was determined as the difference between the EDSS at the time of blood collection and the EDSS at initial diagnosis with a negative ∆EDSS indicating clinical improvement. The ∆gene mRNA value reflects the fold difference between gene expression before and after IVMP with a negative value indicating a decrease in gene expression after therapy. The graphs depict the correlation between both parameters separately for PPMS and SPMS patients with each dot representing one individual patient. The solid line represents the linear regression curve for SPMS, the dotted line for PPMS patients. N = 4–5 (PPMS), N = 8–10 (SPMS). (**B**) The graphs depict the correlation between the EDSS score at the time of blood collection and the ∆gene mRNA value separately for PPMS and SPMS patients with each dot representing one individual patient. The solid line represents the linear regression curve for SPMS, the dotted line for PPMS patients. N = 4–5 (PPMS), N = 9–11 (SPMS). The Spearman correlation coefficient (r) and the level of significance (* *p* < 0.05) are indicated in each graph solely for the analysis of SPMS patients.

Clinical symptoms of MS patients are quantified using the *Expanded Disability Status Scale* (EDSS), which assesses functional impairments such as the ability to walk, work, and eat independently. The EDSS score was determined at the day of blood sampling, and its change since the initial MS diagnosis was calculated as the ∆EDSS (Supplementary Table S2). Initially, we conducted a linear regression analysis of PPMS and SPMS patients to correlate the change in gene expression after IVMP with the ∆EDSS score (Figure 8A). We observed a tendency in SPMS but not PPMS patients that clinical improvement, which means a negative ∆EDSS, was linked to a reduced metabolic gene expression after IVMP (Figure 8A). However, it is noteworthy that the time period corresponding to the ∆EDDS score strongly differs between individual patients, which could represent a confounding factor in this analysis. Therefore, another linear regression analysis was performed to correlate the absolute EDSS score at the day of blood sampling with the change in gene expression after IVMP (Figure 8B). Here, it turned out that the reduction in metabolic gene expression after therapy was most pronounced in those patients with the highest EDSS score (Figure 8B). This correlation was statistically significant for *GLUT*3, *SLC7A*5, and *LDHA* in SPMS patients, whereas no association was observed for PPMS patients. In summary, our analysis provides evidence that the responsiveness of metabolic gene expression to IVMP therapy in T cells correlates with the disease course of SPMS but not PPMS patients.

## 4. Discussion

GCs are used in the management of multiple inflammatory conditions including autoimmunity to keep undesired immune responses at bay. However, despite intensive research over the last decades, their ultimate mode of action has not been completely resolved. This can in part be explained by the fact that GCs act in various ways and on different target cells contingent upon the specific pathogenic condition, as revealed by analyzing genetically engineered mice with cell-type-specific or function-selective GR mutations [1,4,47,48]. In those disease models in which GCs were found to mainly act on T cells, apoptosis induction, a change in motility or chemotaxis, and a reduced production of cytokines, cell adhesion receptors, and cytotoxic molecules were identified as candidate mechanisms of action [3,8,9,11,13]. However, their relative importance remains unknown. Since T cell function is intimately related to immunometabolism [16,17,19], we here tested the hypothesis that this process represents a hitherto unrecognized rheostat for the control of T cell activity by GCs. Our data provide evidence that two synthetic GCs widely used to treat inflammatory disorders such as MS were able to repress genes and processes involved in T cell energy metabolism. Although this conclusion was formally achieved only for Dex and MP, our previous analysis on the regulation of EAE suggests that different GC derivatives generally use similar mechanisms to control inflammation [49].

Our analyses revealed that GCs repress multiple genes involved in different steps of the rapid switch from steady-state OXPHOS to activation-induced aerobic glycolysis, a process that is considered to be important for T cell activation and proliferation and the acquisition of effector functions [15,16]. Some of the identified genes are relevant for the transport of essential nutrients into the cell (*GLUT1* and *GLUT3* for glucose, *SLC1A5* and *SLC7A5* for amino acids), whereas others encode critical enzymes directly involved in the glycolytic pathways (*HK2, ALDOA, LDHA, LDHB*). The suppression of each of these genes can be expected to interfere with the function of T cells by impeding their supply with glucose and metabolites necessary for protein and nucleic acid synthesis. Therefore, it is likely that the metabolic deficit contributes to the impaired cytokine production, cytotoxic activity, and migration of T cells observed after GC treatment [2,3,11]. Functional assays addressing glucose transport and lactate production support our conclusion that the identified gene regulation impedes these metabolic processes in activated T cells. The fact that GCs modulate immunometabolism across species in a largely similar manner makes us confident that this mechanism is indeed highly relevant. Nevertheless, it is noteworthy that there were also differences between murine and human T cells concerning gene regulation by GCs, for instance with regard to *LDHB*. The use of T-cell-specific GR knockout and Bcl-2 tg mice confirmed that the newly identified GC activity was exerted via the GR and independent of the pro-apoptotic activity of these drugs. Our study involving MS patients finally confirmed that the regulation of immunometabolism by GCs also occurs in a clinical setting. Currently, it is unknown whether GCs control metabolic gene expression in a direct manner or whether this effect is indirectly mediated by a master regulator. An interesting candidate for such a function could be FKBP5, a chaperone that is widely expressed in immune cells and strongly upregulated by GCs [50,51,52]. In summary, we conclude that GCs impede the capacity of T cells to rapidly undergo a metabolic switch from steady-state OXPHOS to activation-induced aerobic glycolysis.

Stat1 is involved in signaling pathways activated by interferons, TNFα, and IL-6. A number of studies indicated that GCs interfere with these pathways by suppressing both the upregulation and the phosphorylation of Stat1 in PBMCs and intestinal epithelial cells [39,53,54]. However, the view that Stat1 is directly regulated by GCs is not unchallenged. In macrophages, for instance, it was shown that Stat1 phosphorylation was indirectly repressed by GCs via SOCS1 [55], or even resistant to Dex treatment [56]. Regardless of the exact mechanism, Stat1 is undoubtedly an important target of GCs in many situations including autoimmune diseases. For instance, in MS patients IVMP therapy was reported to reduce pStat1 levels in monocytes and T cells, providing in vivo evidence that GCs may exert some of their anti-inflammatory activity via this mechanism [57]. In our system, the inhibition of Stat1 blunted T cell activation and reduced the expression of several nutrient transporters and glycolytic enzymes, thus unraveling a so far underestimated role of this transcription factor in T cell function. However, GCs were still able to reduce the expression of genes involved in immunometabolism even when Stat1 activity was pharmacologically blocked. Therefore, our results indicate that Stat1 is presumably not involved in mediating the repressive activity of GCs on this process.

Another important transcription factor in many types of immune cells is mTOR. It is contained in the two multi-protein complexes mTORC1 and mTORC2 that coordinate fundamental processes including cell growth, metabolism, and autophagy [58,59]. Control of immunometabolism is mostly achieved via mTORC1, which is inhibited by rapamycin in contrast to mTORC2 [59]. The first evidence has also been obtained that GCs repress mTOR signaling, for instance in tumor cells [60] and regulatory T cells [30]. Moreover, the inhibition of mTOR was found to interfere with the anti-inflammatory activity of GCs in human monocytes and dendritic cells, indicating that it could represent a crucial target of GCs [34]. These findings are in line with the synergistic effect of GCs and calcitriol observed in a mouse model of MS, which was at least partially explained by a reduced mTOR activity in T cells [61]. With that in mind, we hypothesized that GCs regulate immunometabolism by interfering with mTOR function. The inhibition of mTOR by rapamycin had no effect on T cell activation or apoptosis induction but strongly reduced the expression of glucose and amino acid transporters as well as glycolytic enzymes, which is consistent with its role in cellular metabolism. Importantly, Dex was able to modulate T cell activation and apoptosis independently of mTOR activity but failed to repress genes involved in immunometabolism when it was inhibited by rapamycin. The finding that this treatment abolished the ability of GCs to inhibit mTOR phosphorylation in activated T cells too indicates that the control of metabolic gene expression by GCs might be mediated by an interaction of the GR with mTOR signaling. Collectively, our observations unveil the mTOR axis and its control over metabolic processes as an important GC target in T cells.

In vitro models are instrumental in establishing molecular mechanisms but require confirmation in vivo in human subjects. It is therefore crucial that we could demonstrate the principle of metabolic control by GCs in MS patients as well. In line with our in vitro data, the expression of glucose and amino acid transporters as well as glycolytic enzymes declined in the T cells of both PPMS and SPMS patients after IVMP administration. The individual response of each patient was variable, but nevertheless GC treatment reduced the mRNA levels of *GLUT3*, *SLC7A5*, and *LDHA* in many of them. It is unlikely that this effect is due to a preferential induction of apoptosis in highly metabolically active T cells since these cells were found to be resistant to GC-induced cell death [8]. With regard to the observed variability in gene regulation in vivo, it further needs to be considered that the PPMS and SPMS patients who receive IVMP therapy for a long time can develop different degrees of GC resistance [62,63]. In addition, their T cell composition may differ concerning the abundance of effector T cells, a feature which is well known to impact the strength of the GC response [8]. Regardless of these influential factors, our clinical study provides some evidence that metabolic gene expression changes in the T cells of MS patients after IVMP. We are thus confident that immunometabolism is highly relevant as a target of the therapeutic activity of GCs and also impacts clinical features. Namely, we found a weak correlation of the ∆EDSS and a statistically significant correlation of the absolute EDSS with changes in metabolic gene expression. This association was observed for SPMS but not PPMS patients, which could indicate that the former possibly benefit more from cyclic IVMP therapy than the latter.

## 5. Conclusions

Here, we could demonstrate reduced metabolite transport and glycolysis after GC treatment in murine and human T cells as well as MS patients. Moreover, our results indicate that these effects are presumably mediated by the GCs’ impact on the mTOR pathway. Whereas other processes such as reduced cytokine secretion or an altered migratory capacity have already been claimed to account for the anti-inflammatory activity of GCs in the past, our findings now suggests that metabolism is another rheostat of T cell function influenced by these drugs. Further work is required to ultimately unravel the relative contribution of the different mechanisms to the therapeutic efficacy in each condition, but already now we are inclined to believe that our observation will be instrumental in developing improved GC-based therapies.

## Data Availability

Data and material are available from the corresponding authors on reasonable request.

## Supplementary Materials

The following supporting information can be downloaded at: https://www.mdpi.com/article/10.3390/cells12030450/s1, Figure S1: Kinetics of metabolic gene expression in murine T cells after Dex treatment in vitro; Figure S2: Repression of T cell activation, apoptosis, and glucose import in human peripheral CD8^+^ T cells by Dex in vitro; Figure S3: Exemplary flow cytometric analysis of in vitro cultured human peripheral blood T cells for Stat1 activation; Figure S4: Exemplary flow cytometric analysis of in vitro cultured human peripheral blood T cells for mTOR activation; Figure S5: Comparison of metabolic gene expression in human peripheral blood T cells from MS patients before and after IVMP therapy; Table S1: Primer sequences; Table S2: Patient characteristics.
