# Supplementary material for: T Cell Energy Metabolism Is a Target of Glucocorticoids in Mice, Healthy Humans, and MS Patients
Source: Cells. 2023 Jan 30;12(3):450. doi: 10.3390/cells12030450 (PMC9914408; doi:10.3390/cells12030450)
Supplement: Supplementary file 1 [file cells-12-00450-s001.zip › cells-2155943-supplementary.pdf]

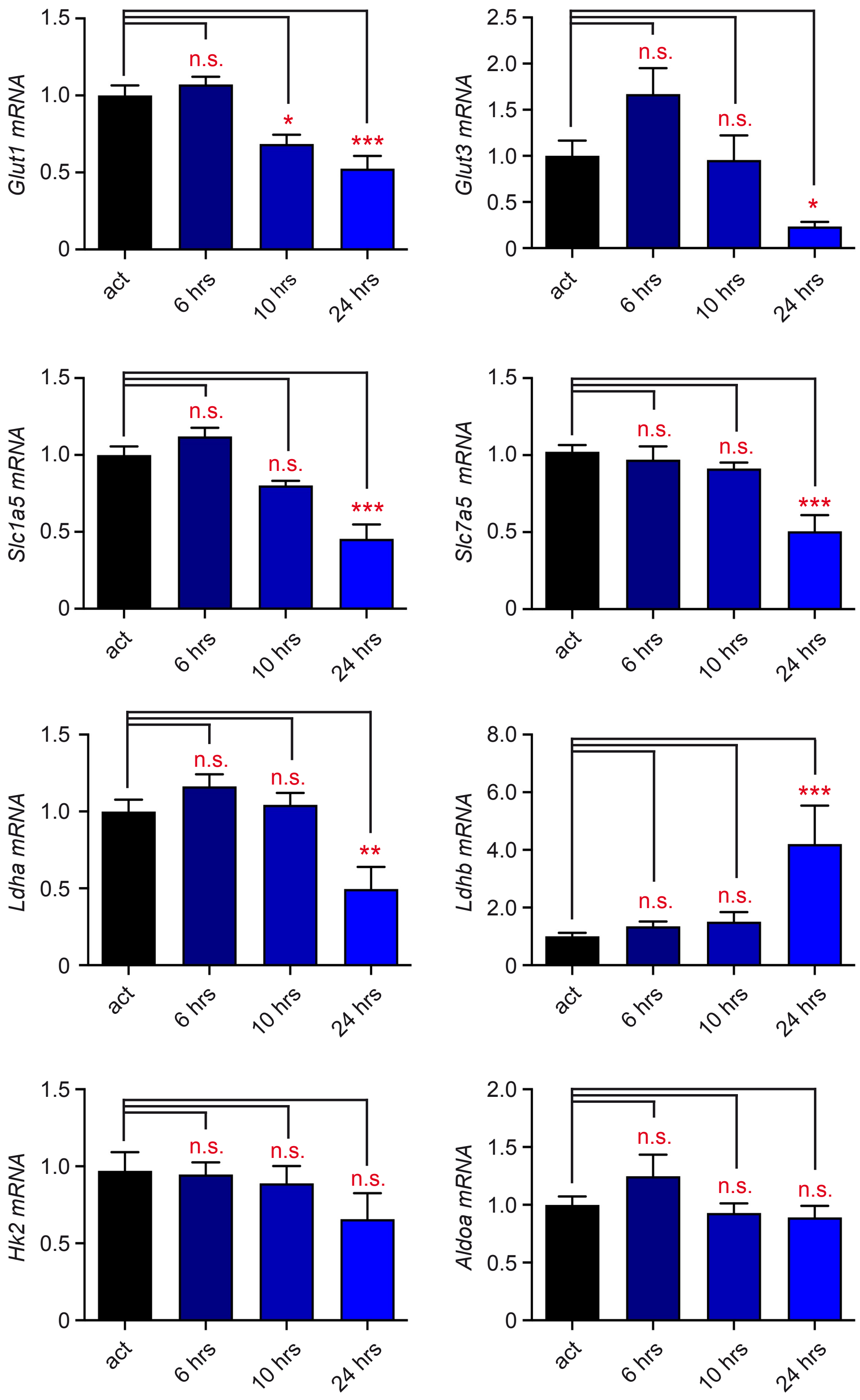

Supplementary Figure S1. Kinetics of metabolic gene expression in murine T cells after Dex treatment *in vitro*. T cells were purified from spleen and lymph nodes of C57BL/6 mice and stimulated with mCD3ε and mCD28 antibodies for 24 hrs (act). To some samples, 10^{-6} M Dex was added during the last 6, 10 or 24 hrs of incubation. Gene expression was determined by RT-qPCR. N=7-30. Relative mRNA levels were calculated by normalization to the housekeeping gene *Hprt*. Expression in activated T cells was arbitrarily set to 1. Values are depicted as the mean ± SEM, statistical analysis was done by One-way ANOVA followed by a Multiple Comparisons test. Levels of significance: *: $p < 0.05$; **: $p < 0.01$;***: $p < 0.001$; n.s.: $p > 0.05$.

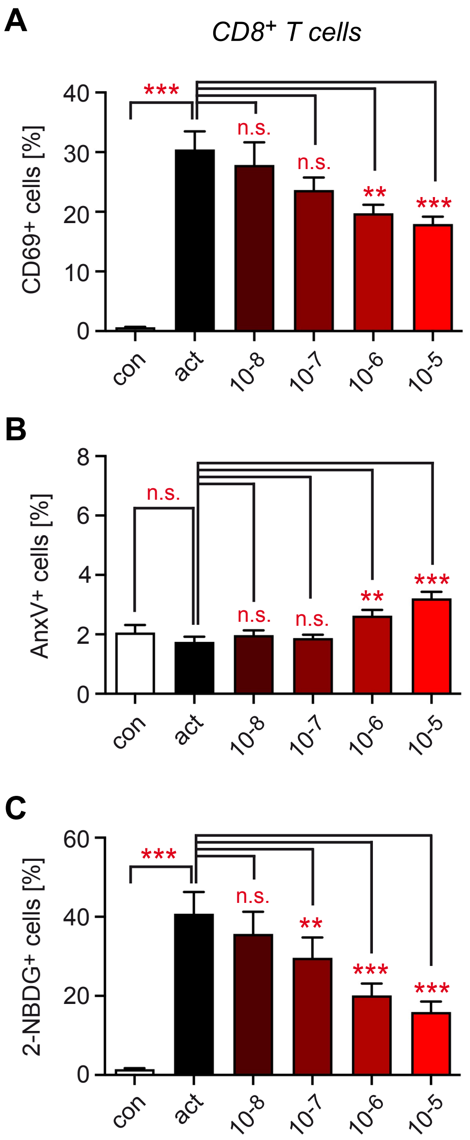

Supplementary Figure S2. Repression of T cell activation, apoptosis and glucose import in human peripheral blood $CD8^+$ T cells by Dex *in vitro*. T cells were purified from buffy coats of healthy human donors and stimulated using hCD3ε and hCD28 antibodies for 20 hrs in the absence (act) or presence of 10^{-8} to 10^{-5} M Dex. Unstimulated T cells served as a control (con). The percentages of **(A)** $CD69^+$ and **(B)** $AnxV^+$ $CD3^+CD8^+$ T cells were determined by flow cytometric analysis; N=17/12. **(C)** Glucose import was determined by incubating the cells for 1 hr with fluorescently labelled 2-NBDG and then measuring the percentage of $CD3^+CD8^+$ T cells positively stained for 2-NBDG by flow cytometry; N=15. All values are depicted as the mean ± SEM. Statistical analysis was performed by One-way ANOVA followed by a Multiple Comparisons test. Levels of significance: *: $p < 0.05$; **: $p < 0.01$; ***:$p < 0.001$; n.s.: $p > 0.05$.

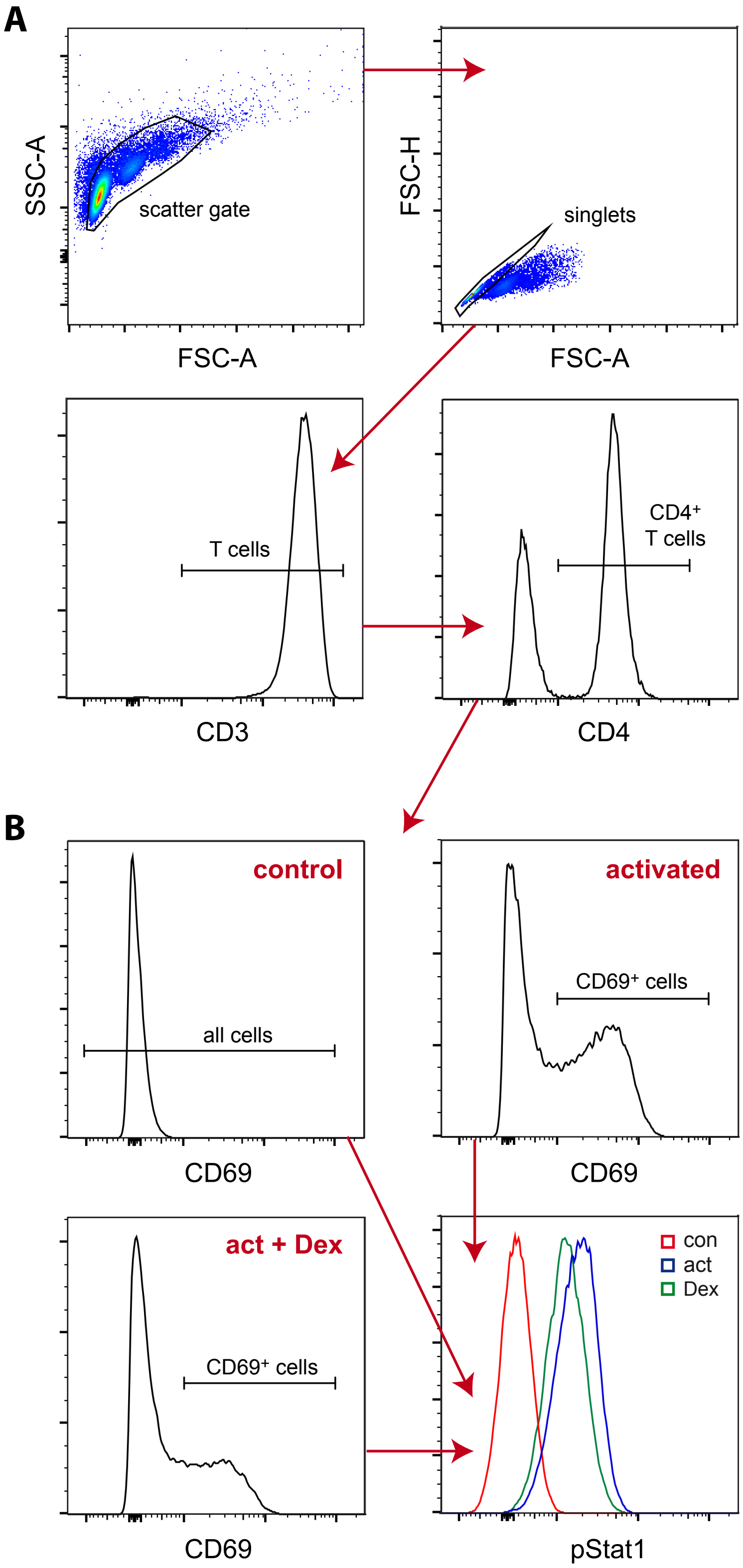

Supplementary Figure S3. Exemplary flow cytometric analysis of *in vitro* cultured human peripheral blood T cells for Stat1 activation. **(A)** Relevant cells were first defined on the basis of their forward and sideward scatter, followed by gating on singlets. $CD4^+$ T cells were then identified using antibodies recognizing CD3 and CD4. **(B)** CD69 was used as a marker to characterize activated T cells after costimulation for 20 hrs in the absence (act) or presence of 10^{-5} M Dex or being left untreated (con). pStat1 levels are depicted as overlay histograms either gated on all cells (con) or $CD69^+$ cells (act/Dex).

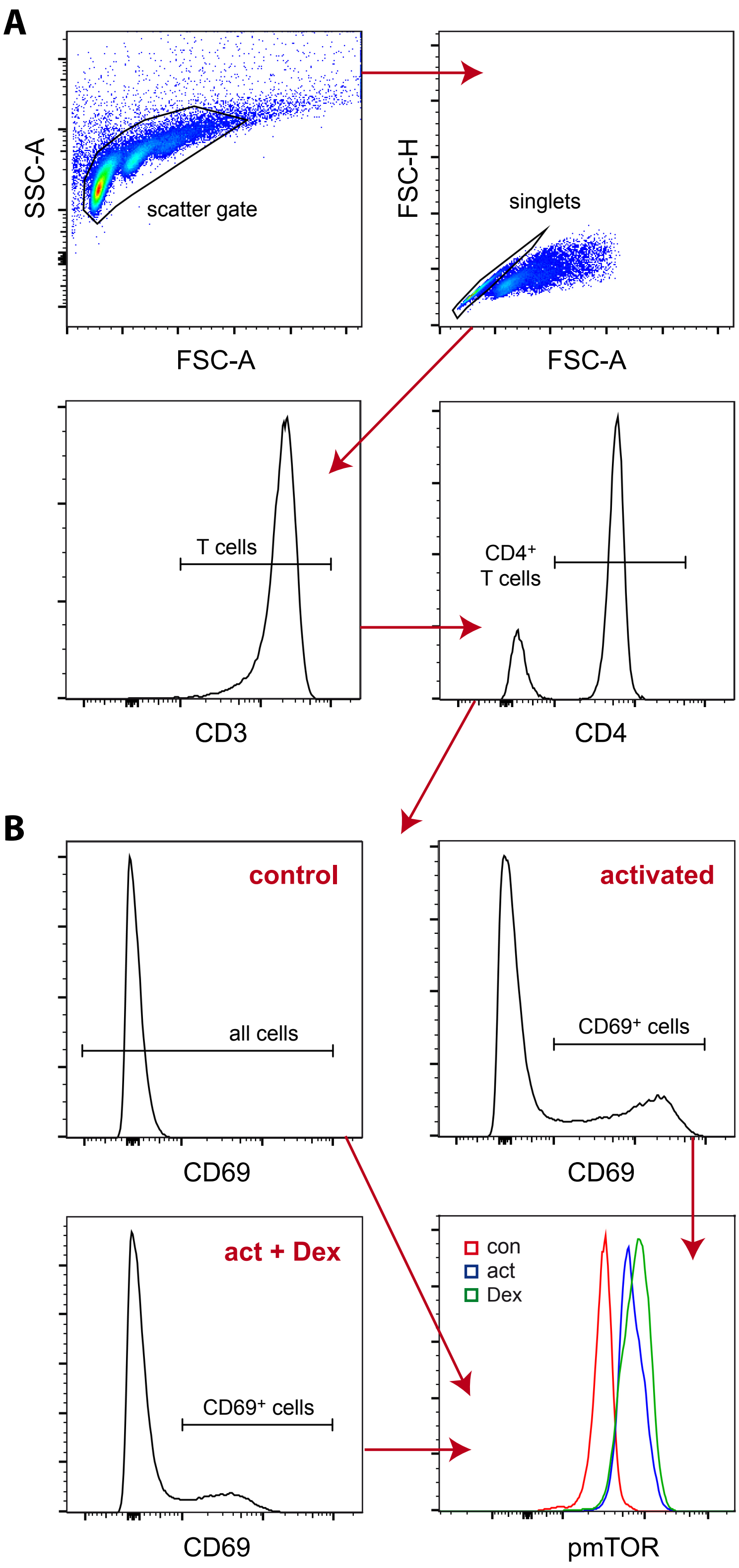

Supplementary Figure S4. Exemplary flow cytometric analysis of *in vitro* cultured human peripheral blood T cells for mTOR activation. **(A)** Relevant cells were first defined on the basis of their forward and sideward scatter, followed by gating on singlets. $CD4^+$ T cells were then identified using antibodies recognizing CD3 and CD4. **(B)** CD69 was used as a marker to characterize activated T cells after costimulation for 20 hrs in the absence (act) or presence of 10^{-5} M Dex or being left untreated (con). pmTOR levels are depicted as overlay histograms either gated on all cells (con) or $CD69^+$ cells (act/Dex).

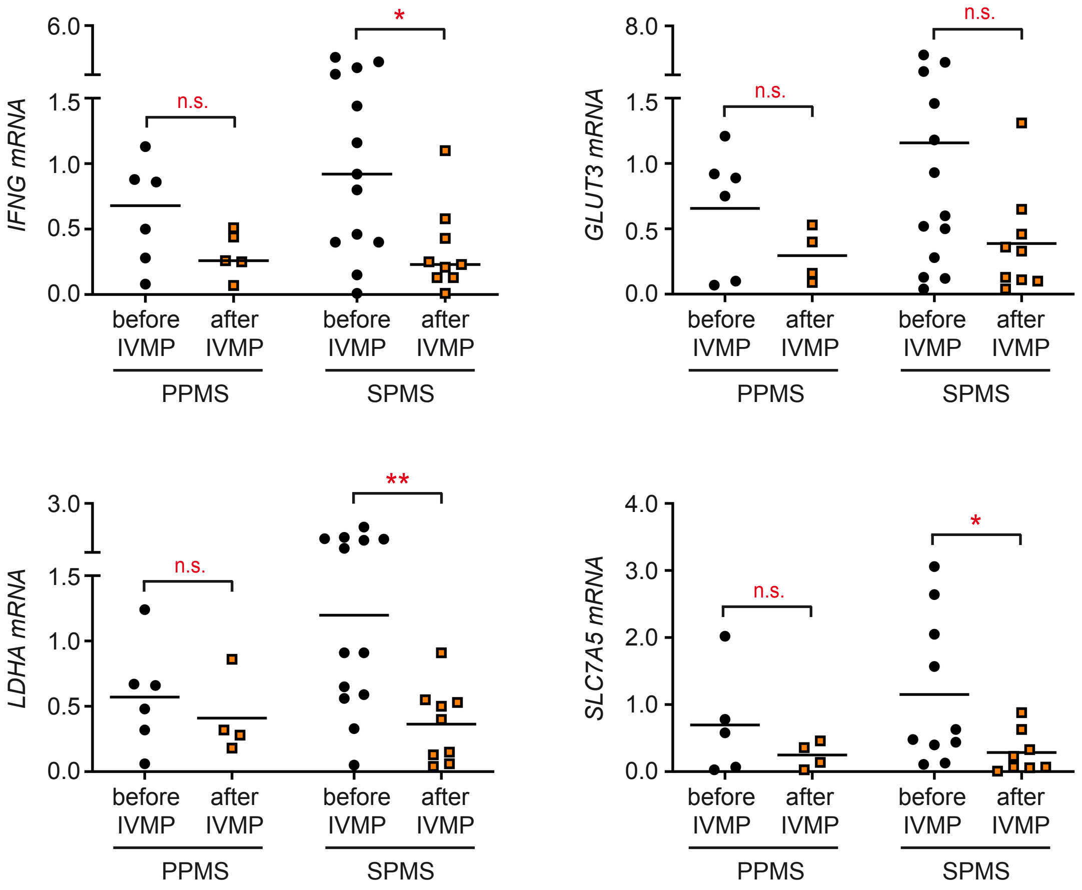

Supplementary Figure S5. Comparison of metabolic gene expression in human peripheral blood T cells from MS patients before and after IVMP therapy. T cells were purified from blood samples collected either from PPMS or SPMS patients immediately before IVMP therapy and 24 hrs later. Gene expression was determined by RT-qPCR and relative mRNA levels were calculated by normalization to the housekeeping gene 18SRNA. N=4-6 (PPMS), N=8-13 (SPMS). Each dot corresponds to one patient. The data are identical to Figure 8C except that they are separately depicted for each disease subtype. Statistical analysis was performed by unpaired t-test. Levels of significance: *: $p < 0.05$; **: $p < 0.01$;n.s.: $p > 0.05$.

Supplementary Table S1. Primer sequences

Gene	Forward primer	Reverse primer
mAldoa	*5'-CAG ATG GGT CCA GCT TCA AC-3'*	*5'-TGC TTT CCT TTC CTA ACT CTG TC-3'*
mGlut1	*5'-AGC ATC TTC GAG AAG GCA GG-3'*	*5'-ACA ACA AAC AGC GAC ACC AC-3'*
mGlut3	*5'-CTC TTC AGG TCA CCC AAC TAC GT-3'*	*5'-CCG CGT CCT TGA AGA TTC C-3'*
mHk2	*5'-GCC TCG GTT TCT CTA TTT GGC-3'*	*5'-ATA CTG GTC AAC CTT CTG CACT-3'*
mHprt	5'-GTC CTG TGG CCA TCT GCC TA-3'	5'-GGG ACG CAG CAA CTG ACA TT-3'
mLdha	*5'-AGT AAG TCC TCA GGC GGC TA-3'*	*5'-GGA CTT TGA ATC TTT TGA GAC CTT G-3'*
mLdhb	*5'-AAA GGC TAC ACC AAC TGG GC-3'*	*5'-GCC GTA CAT TCC CTT CAC CA-3'*
mSlc1a5	*5'-CGC TAT CGT CTT TGG TGT GG-3'*	*5'-GGG TGC GTA CCA CAT AAT CC-3'*
mSlc7a5	*5'-GGG GAA GGA CATG GGA CAAG-3'*	*5'-ATA GTT CCAT CCT CCG TAG GCG-3'*
h18SRNA	*5'-TCC AGG TCT TCA CGG AGC TTG TT-3'*	*5'-GGA TGT AAA GGA TGG AAA ATACA-3'*
hALDOA	5′-TGT CCA TGG CTA TGG CCT TTT-3′	5′-TGG TAG TAG CAA GTT CCT TTC CTA-3′
hGLUT1	5′-TGG CAT CAA CGC TGT CTT CT-3′	5′-AGC CAA TGG TGG CAT ACA CA-3′
hGLUT3	5′-GGA CGT GGA GAA AAC TTG CTG-3′	5′-TCA GAG CTG GGG TGA CCT TC-3′
hHK2	5′-CCT CCC CTC TCG CGT CT-3′	5′-AGA GAT ACT GGT CAA CCT TCT GC-3′
hIL2	*5′-TTT ACA TCG CCA AGA AGG CCA-3′*	*5′-GCA CTT CCT CCA GAG GTT TG-3′*
hIFNG	*5′-CTG TAA CTG CCA GGA CCC AT-3′*	*5′-TCT GTC ACT CTC CTC TTT CCA-3′*
hLDHA	5′-ACG TGC ATT CCC GAT TCC TT-3′	5′-AAC AGC ACC AAC CCC AAC AA-3′
hLDHB	5′-GCC TTC TCT CTC CTG TGC AA-3′	5′-CCT CTT CTT CCG CAA CTG GT-3′
hSLC1A5	5′-AGT GGG CTT GGC GCT G-3′	5′-TGG AAG GGA AGA TAT TTC TCG CA-3′
hSLC7A5	5′-TCA TCG CAG TAC ATC GTG GC-3′	5′-TGA GCA GCA GCA CGC AG-3′

Supplementary Table S2. Patient characteristics

	PPMS	SPMS	total
Number of patients	12	17	29
Age (years, ±SD)	60.5 (12.5)	65.8 (10.1)	63.6 (11.5)
Females (number, %)	6 (50)	10 (58.8)	16 (55.2)
EDSS Score (mean ± SD)	5.0 (1.2)	6.1 (1.1)	5.7 (1.2)
ΔEDSS Score (mean ± SD)	0.21 (0.76)	0.17 (0.96)	0.18 (0.87)
Disease duration (years, ±SD)	10.9 (9.4)	27.5 (13.3)	21.7 (14.5)
IVMP therapy (years ± SD)	3.3 (3.4)	3.5 (4.2)	3.4 (3.9)
